# Using the Coefficient of Conformism of a Correlative Prediction in Simulation of Cardiotoxicity

**DOI:** 10.3390/toxics13040309

**Published:** 2025-04-16

**Authors:** Alla P. Toropova, Andrey A. Toropov, Alessandra Roncaglioni, Emilio Benfenati

**Affiliations:** Department of Environmental Health Science, Istituto di Ricerche Farmacologiche Mario Negri (IRCCS), Via Mario Negri 2, 20156 Milano, Italy; andrey.toropov@marionegri.it (A.A.T.); alessandra.roncaglioni@marionegri.it (A.R.); emilio.benfenati@marionegri.it (E.B.)

**Keywords:** cardiotoxicity, human ether-a-go-go-related gene (hERG) blocker compounds, QSAR, Monte Carlo method, coefficient of conformism of a correlative prediction (CCCP), external validation, CORAL software

## Abstract

The optimal descriptors generated by the CORAL software are studied as potential models of cardiotoxicity. Two significantly different cardiotoxicity databases are studied here. Database 1 contains 394 hERG inhibitors (pIC50) and external 200 substances that are potential drugs, which were used to confirm the predictive potential of the approach for Database 1. Database 2 contains cardiotoxicity data for 13864 different compounds in a format where active is denoted as 1 and inactive is denoted as 0. The same model-building algorithms were applied to all three databases using the Monte Carlo method and Las Vegas algorithm. The latter was used to rationally distribute the available data into training and validation sets. The Monte Carlo optimization for the correlation weights of different molecular features extracted from SMILES was improved by including the conformity coefficient of the correlation prediction (CCCP). This improvement provided greater predictive potential in the considered models.

## 1. Introduction

There is no doubt that cardiovascular diseases are some of the most important medical problems globally. Accordingly, drug monitoring for cardiotoxicity should also be considered a very important medical problem. Data on cardiotoxicity are needed for the development of a wide range of new drugs [1]. The human potassium channel gene (hERG) plays an important role in regulating heart rate, and data on cardiotoxicity associated with hERG inhibition by drugs and environmental chemicals provide essential information for medicinal chemistry. Enhancing cardiotoxicity data in direct experiments is large-scale, expensive, and virtually impossible. Therefore, the use of in silico models can help to reach this endpoint. For instance, to define the hierarchy of molecules in the early stages of new drug development and minimize the risks of using new pharmaceutical agents, computational approaches are used to predict the hERG-blocking potential of new drug candidates. Indeed, quantitative structure–property/activity relationships (QSPRs/QSARs) for the cardiac toxicity of organic hERG blockers are reported in the literature [2,3,4,5].

A very complex model requires too much knowledge and too many skills. Convenient models that do not call for significant intellectual effort (i.e., economy of thinking) are therefore desirable, extracting information from data already available, such as effect data associated with chemical structures. However, chemical information can be described and processed in many ways, depending on the chemical structure format. It is interesting to compare the practical applications of InChI (International Chemical Identifier) and SMILES (Simplified Molecular Input Line Entry System), which are common formats used to describe the structure of a substance. According to SCOPUS, citations of works using InChI for QSPR/QSAR analysis are only 2% of those using SMILES in QSPR/QSAR analysis [6,7].

The CORAL software (http://www.insilico.eu/coral, accessed on 11 April 2025) requires only SMILES and numerical data on an endpoint to build a model. Therefore, while the approach used here is fairly convenient, it should be noted that two innovations are applied to the construction of the cardiotoxicity models described here. First, the Conformity Coefficient of Correlative Prediction (CCCP) [8] was used to improve the efficiency of the Monte Carlo method for model generation. Second, the Las Vegas algorithm [8,9] was used to select a prospective split of the available data into training and validation sets. It is likely that this is the first time that these steps have been applied to the construction of a cardiotoxicity model.

The aim of the study to attempt to assess the influence of these new characteristics [10,11] on the predictive potential of cardiotoxicity models in order to properly develop successful models.

## 2. Results

### 2.1. Database 1

Cardiotoxicity models were developed using two databases. The first database contains data on 394 organic molecules with a range of pLD50 values (−3.64, 2.00) and was used for regression models [1]. 

Two approaches were compared, and their differences lie in the use of the CCCP parameter. Table 1 gives the statistical characteristics of models obtained using target function T_1_, and Table 2 shows the results obtained using target function T_2_, which includes the CCCP parameter. Comparing the results of the two algorithms, the predictive potential of models using target function T_2_ is better than that of models using target function T_1_. For instance, the R^2^ of the validation set of the three models (using the three partitions) is always below 0.7 in the case of target function T_1_, but always above 0.7 for target function T_2_. Similarly, the R^2^ of the calibration set is always below 0.78 for target function T_1_ but is always above 0.81 for target function T_2_. In general, improvement is clear for all the statistical parameters in Table 1 and Table 2. 

**Figure 1:**
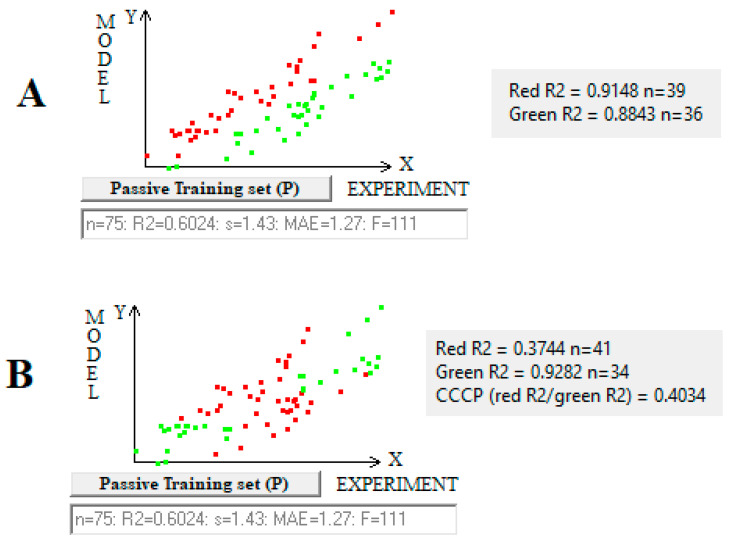
The comparison of geometrical interpretations for (**A**) the division of the total correlation clusters into two sub-clusters caused by the influence of IIC or CII and (**B**) the graphical interpretation of the opponents and supporters of the correlation.

Figure 1 compares the correlation coefficients for experimental and predicted pIC50 values. It is important to note the difference between the red/green division in cases A and B (Figure 1). The red/green division in the first case is based on the quality assessed by calculating the difference between the “experimental value and the calculated value” of individual points, whereas in the second case, the red/green division is based on the difference between the correlation coefficients obtained by removing all substances one at a time. It can be seen that the configurations (red/green) of the geometric arrangement of the image points in cases A and B in Figure 1 differ significantly. When considering the set of points forming the geometric image of the correlation, special subsets can be defined. Points for which the forecast is accurate or overestimated are shown in Figure 1A in green. Points for which the forecast is underestimated are shown in Figure 1A in red. Based on this division of points, the index of the ideality of correlation (IIC) is calculated [8]. When calculating the correlation intensity index (CII) and the CCCP, other subsets are considered. Their definition is as follows. If the removal of a point is accompanied by an increase in the correlation coefficient, this point is classified as being an opponent to the correlation. This means that this substance has a detrimental role from a statistical point of view. These points are indicated by red in Figure 1B. If the removal of a point is accompanied by a decrease in the correlation coefficient, this point is classified as a supporter of correlation. In other words, these substances contribute more than the others to the good quality of the model. These points are indicated in green in Figure 1B. CII is the sum of the contributions of all opponents of correlation. CCCP is the ratio of the sum of the effects of all opponents of correlation to the sum of all supporters of correlation.

The IIC, CII, and CCCP were tested as values capable of influencing the predictive potential of models [8].

**Figure 2:**
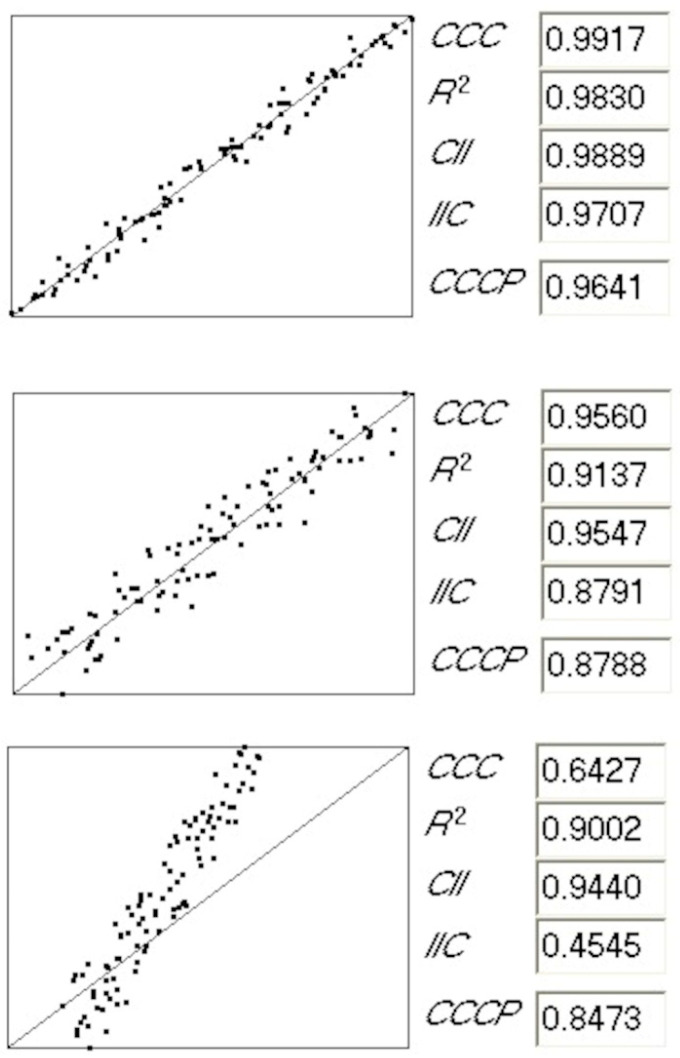
Comparison of the statistical parameters obtained for three regression cases.

There are differences and relationships in the values obtained for the various parameters applied to different cases. These are presented in Figure 2. For instance, we observe that the concordance correlation coefficient (CCC) and IIC are sensitive to the change in the slope of the regression, and their values are low for the regression presented at the bottom of the figure. Conversely, the decreases in other parameters are similar when there is a relative spread of the values regarding the perfect regression (such as for the regression in the middle of the figure) and when the slope is affected (such as for the regression at the bottom of the figure).

**Figure 3:**
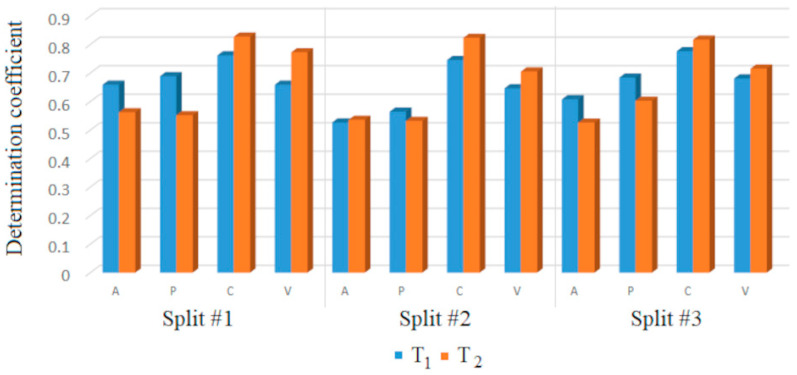
Comparisons of the determination coefficients for experimental and predicted pIC50 in the active (A), passive (P), calibration (C), and validation (V) sets.

The Las Vegas algorithm [8,9] selects the best Monte Carlo model from a group of attempts to build the model. The selection is based on the statistical quality of the model for the calibration set. The most meaningful statistical parameters are those associated with the model once it is fully developed, represented by the calibration set. The model is ready only when the modeling parameters are optimized, i.e., at the last step of the modeling process, which is carried out with the calibration set. The previous modeling steps, as implemented for the values for the active and passive training sets, are only preliminary, and the model is not mature. Thus, the statistics for the active training and passive training sets are less important for the evaluation of the final model. Table 1 and Table 2 and Figure 3 demonstrate that the CCCP is useful in the process of Monte Carlo optimization. Figure 3 shows the correlation coefficients obtained with the different sets. 

**Figure 4:**
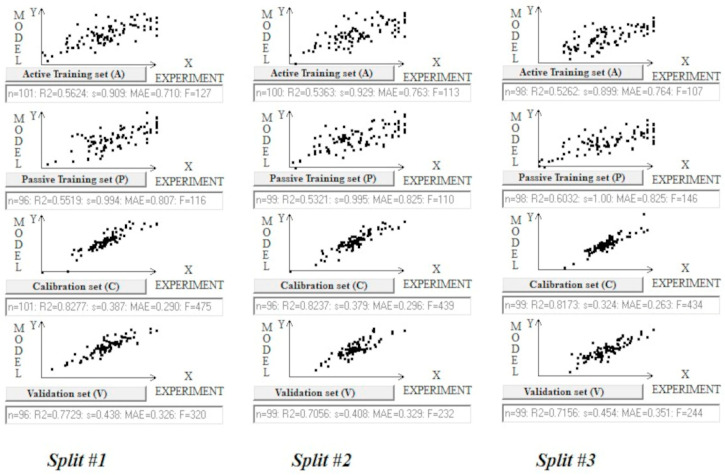
Graphical representation of models for random splits obtained using target function T_2_.

Figure 4 illustrates the correlations for the different subsets. It is clear that in the calibration step, the model is mature and there is closer alignment between the calculated and experimental values than in the active and passive training sets, which show a much larger spread of values.

The comparison of the model based on the T_2_-optimization shows that our results are comparable with the statistical quality of other models with the same endpoint, as reported in Table 3. In a previously published study, the results were good [12], but they utilized a much smaller dataset. The values reported in [12], with R^2^ 0.81, are closely comparable with the *R*^2^ of the calibration set, which is always above 0.81, with a mean value of 0.82. Thus, the model we presented here gave slightly better results than the best model in Table 3. In addition, our model utilizes a larger dataset, which is always an advantage since it provides a larger, and therefore more robust, basis, providing an increased applicability domain. There are other advantages. An advantage of our model compared to others is that it is quite simple in its general approach and implementation. Indeed, the CORAL software (http://www.insilico.eu/coral, accesed on 11 April 2025) simply needs SMILES as input, and there is no need to calculate molecular descriptors. It is useful to compare the results of our model with those in [1], because in this case, the comparison examines the exact same sets of substances (the apparently different number of substances indicated in Table 3 is due to the fact that we identified duplicates in the original dataset). The model described in [1] implies multiple very complex systems: docking simulation and three-dimensional QSAR models. Not only is the strategy complex, requiring not only one algorithm, but the components of this combined system are quite demanding, since a docking simulation is needed and models use three-dimensional descriptors. Thus, the system, the algorithms, and the molecular descriptors are quite complex. Conversely, our tool does not require the integration of multiple models, does not require complex algorithms and the calculation of three-dimensional descriptors is not necessary. In reality, molecular descriptors are not needed at all, since the software simply and directly uses SMILES. Thus, overall, our model offers several aspects of innovation that provide better results when compared using the same dataset. 

### 2.2. Database 2

The second database was used to assess the statistical utility of the approach used in the case of classification models. For this purpose, three classification models were constructed using semi-correlations for the hERG inhibitory capacity of a large array of compounds taken from the literature [17]. From the entire dataset, all active compounds were considered together with an equal number of inactive compounds. The total number of considered compounds was 13,846 (endpoint values 0 for non-toxic compounds and 1 for toxic ones). 

Table 4 contains the predictive potential of models based on semi-correlations obtained with target function T_1_ and target function T_2_. Again, it can be observed that the T_2_ provides better models, even if the results obtained with target function T_1_ are equally as good. Also in this case, as we previously highlighted in the case of regression model, the mean values are those obtained using the calibration set, which are related to the optimized model. These values should then be compared with those obtained using the substances in the validation set, which are not used in the model development. These represent the application of the model to new substances, and ideally, the difference in the statistical values for the calibration and validation sets should be small. This is demonstrated in Table 4, indicating that the model has good statistical values regarding both its robustness and its predictivity.

Table 5 contains a comparison of the statistical quality of models presented in the literature and models based on semi-correlations built using the CORAL software [18].

The only model with results comparable to those presented here can be those observed in [22]. However, that model utilized only 7889 compounds, whereas the model suggested in this study used 13,846 compounds and is thus expected to have a broader applicability domain. 

The case of the comparison of the results of the different models in Table 5 is quite similar to the case we discussed previously regarding results comparisons in regression models. There are advantages due to the fact that our model has a larger dataset compared with the dataset of the best other model in the table, thus representing a more robust model. We compare our results with those obtained in the model outlined in [17] because we used the same dataset and thus the comparison is more appropriate. The accuracy of the model in [17] is slightly worse than that which we obtained, while the specificity is the same. However, this representation is misleading. We observed that the sensitivity is only 0.67 in the other study, while it is >0.99 in our case. Thus, the statistics are much better in our case. This is also due to our strategy of splitting the set of inactive substances into different sets, repeating the modeling approach; this solves the issue of unbalanced datasets. The model in [17] used a much more complex algorithm to support vector machines and hundreds of molecular descriptors calculated using different programs. Conversely, our algorithm is much simpler and does not use molecular descriptors at all. Thus, all these points represent useful innovative aspects of the modeling approach presented here. 

## 3. Discussion

The CORAL software has been used to build QSPR/QSAR models for over ten years by many institutes [28,29,30,31,32,33,34,35,36,37,38,39,40,41,42,43,44,45,46,47,48,49,50,51,52,53,54,55,56,57,58]. The basic idea used to develop this software is the use of SMILES to represent molecular structures. Most of the previously used QSPR/QSAR models were based on the use of a molecular graph to represent molecular structures, in which the vertices represent different chemical elements and the edges represent covalent bonds [59,60]. 

Both of the aforementioned options for representing the molecular structure have their advantages and disadvantages; however, the main thing is that these representations of molecular structures are far from identical, and therefore, in principle, can complement each other. The CORAL software makes it possible to construct models based on SMILES or the representation of the molecular structure in the form of a molecular graph, as well as through hybrid representation with the involvement of molecular features expressed by SMILES attributes together with invariants of molecular graphs in the modeling process [61,62,63,64].

In addition to the possibility of constructing hybrid descriptors as above indicated, specific molecular features that are characteristic of the molecular system as a whole were considered for QSPR/QSAR modeling. These are the global attributes of SMILES, such as BOND, NOSP, and HALO. The BOND is a code of covalent bonds. The NOSP is a code that represents configurations of nitrogen, oxygen, sulfur, and phosphorus in a molecular system.

SMILES can serve as a basis for developing other variants of molecular structure information that are capable, in principle, of somehow intersecting with complex biochemical features of molecular structures that determine the biological activity of a substance. These may be combinatorial features, such as contributions of individual atoms or proportions of pairs of atoms [11].

The fragments of local symmetry (FLS) examined in this study are partially related to mathematical symmetry. On the one hand, they have a significant level of symmetry, but in a topological sense, applied to local situations in the molecule, and not to the whole structure. On the other hand, this fact indicated that the molecular fragments represented by FLS are not related to the traditional symmetry concept, which is global. However, FLSs can improve the predictive potential of QSAR models. This is confirmed in the study.

In addition to this kind of representation, there are other possibilities to influence the results of stochastic optimization. These are the statistical criteria of the forecast potential, namely, the correlation ideality index, the correlation intensity index, and the coefficient of conformism for correlative prediction.

The basic principles and expectations when using stochastic modeling with the Monte Carlo approach via the CORAL program (http://www.insilico.eu/coral) are as follows.

1. Any QSPR/QSAR model can be affected by the presence of certain substances in a given (sub)set, thus it is a random event. Therefore, considering one distribution for the training and the validation sets is not enough for a robust assessment of the predictive potential of the method used; it is necessary to consider several random (non-identical) splits in the training and the validation sets.
2. Even when considering a single split into training and validation sets, multiple runs of the stochastic Monte Carlo simulation process will yield different values of the statistical characteristics of the training and validation sets. In this case, the important and necessary information for the correct assessment of the predictive potential of the method obtained is the dispersion of the statistical values for the training and validation sets.
3. This dispersion is not necessarily associated with an error (limitation of the predictive potential) of the considered method; there may be cases of special influence of the IIC and CII factors, which divide the correlation cluster into two sub-clusters for the training set, as we showed in Figure 1.
4. To strengthen the statistical reliability of the selected divisions in training and validation sets, the Las Vegas algorithm was used to obtain the divisions with minimal statistical defects. The essence of the specified stochastic process (the Las Vegas algorithm) is the construction of structured training and validation sets. The structured training set includes passive and active training sets accompanied by a calibration set. The statistical defect of each of the specified sets is the sum of the statistical defects of the SMILES in the abscissa of the model.
5. Ideally, a good model yields similar statistical parameters for the training and validation sets.

The results of the computational experiments considered here confirm the relevance of the above points, although this type of study should be replicated for different endpoints.

This study was planned as a means of testing the ability of local symmetry fragments in cooperation with the CCCP to improve the predictive potential of the models. The comparison of Table 1 and Table 2 demonstrated that the CCCP results in significant improvement in the statistical quality of the models. The corresponding computational experiments without the correlation weights of the FLS have shown that without these, the models are inadequate. Additional studies with different endpoints may explore whether this is true in other cases too.

At present, the study of IIC and CII has shown that the potential for using IIC is greater than that for CII, even though the combined use of IIC and CII may be beneficial. It is possible to manage the process of cooperation between IIC and CII by using coefficients similar to F_1_–F_3_ used in Equations (3) and (4).

Similar studies can be conducted for the new criterion of the predictive potential of the CCCP considered here. Advantages of this criterion in terms of improving the stochastic processes used here for constructing models, both in terms of the Monte Carlo method and in terms of the Las Vegas algorithm, may require additional verification. The study of both the cooperative application of the considered criteria of the forecast potential and their individual capabilities is broad in scope. Obviously, this not only requires experimental implementation but also theoretical understanding. From this point of view, it should be noted that the main advantage of the IIC is its ability to take into account both the correlation coefficient and the mean absolute error (MAE) and/or the root mean squared error (RMSE). To assess the correlation intensity (to calculate CII), other parameters of abstract correlation are used that do not depend on the dispersion of correlation clusters in the “experiment forecast” or “experiment calculation” coordinates (i.e., they do not depend on RMSE and MAE). In fact, optimizing the correlation weights can be interpreted as making a “generalized” decision that affects all compounds (SMILES), not just the training and unseen training sets. This is similar to making a generalized decision through in bicameral legislature in a state parliament. A bicameral legislature is used to avoid a biased decision that is preferred by a particular group of representatives in a parliament. Similarly, two groups of substances that have an unequal influence on the final decision, used separately as training sets and unseen training sets, can help to avoid the biased decision that is preferred by visible substances. The “protests” (substances with an opposite behavior) underlying the calculation of the CII allow us to consider the correlation to be a structure similar to the above-mentioned bicameral legislature. This allows us to compare different correlations using this rule: the smaller the sum of protests [8], the higher the correlation value. The calculation of the CII and the CCCP have a similar basis, which is the so-called “protest” [8]. However, the main difference between the CCCP and the CII is that not only “protests” are taken into account but also the opinion of the supporters of the correlation, that is, the compounds (SMILES) that have a negative protest value. This apparently results in an advantage of the CCCP compared to the CII, because taking into account all opinions is more balanced and yields a higher quantity of information about various phenomena.

In silico simulation can be used as a method of cognition functions by feedback, i.e., any model should be verifiable. For the considered models, an interested user can carry out verification conveniently. It is necessary to download the CORAL program and run it using the splits available from the Supplementary Materials of this study. For QSPR/QSAR simulation by means of stochastic variation in model parameters, a necessary condition is the reproducibility of the results (the values of statistical parameters for the considered partitions). As shown, the reproducibility of the forecast potential is observed with good statistical quality (0.73 ± 0.03 for the determination coefficients for the validation samples). Thus, the proposed modeling concept can be accepted as a convenient tool for QSPR/QSAR analysis. 

In principle, the important points related to the development and use of models are the universality and the possibility of standardization. Universality is understood as the ability of the approach to serve various classes of compounds in QSPR/QSAR analysis. heckedStandardization is the ability to determine the essential criteria that guarantee a good level of predictive potential. In terms of universality, the proposed approach can be easily transposed into a modeling tool based on eclectic data using the so-called quasi-SMILES [10]. In terms of standardization, the above-mentioned IIC and CII, as well as the new parameter CCCP, can be used.

To evaluate the proposed approach, a validation of the model obtained for split 1 was performed. For this purpose, the corresponding SMILES-based descriptors were calculated for 200 compounds outlined in [15]. This validation confirmed the predictive potential of the model obtained for split 1. The technical details of this validation are presented in the Supplementary Materials (Table S2).

The above demonstrate that the new possibilities for constructing the stochastic models discussed here, namely (i) the CCCP and (ii) the Las Vegas algorithm, look quite promising.

We plan to conduct corresponding studies in the future, expanding the study scope by considering further cases, including traditional SMILES, organic and inorganic substances, and peptides and nanomaterials using quasi-SMILES. 

## 4. Materials and Methods

Regression model

Database 1

The first database is for regression models. The numerical data on cardiotoxicity expressed in logarithmic units (pIC50) were taken from the literature [1]. Twelve duplicates were detected in the database. After removing the duplicates, the total number of compounds was 394. These were randomly divided into three partitions to produce three models. Each partition contained a structured training set containing the active (A), passive (P), and calibration (C) training sets; in addition, a validation (V) set was used to evaluate the results of the model using new substances (invisible during the construction of the model).

Descriptors

The descriptors applied were calculated as follows:

(1) $$\;DCW\left(T,N\right)=\sum \;\left\{\;\right.CW\left(S_{k}\right)+CW\left({SS}_{k}\right)+CW\left({SSS}_{k}\right)\left.\;\right\}+\sum CW(FLS)+\sum CW\left(APP\right)$$

*S_k_* is a SMILES atom, i.e., a single symbol or a group of symbols (‘C’, ’O’, ‘N’, etc.) that should be considered a united system (‘Cl’, ‘@@’, %11, etc.); *SS_k_* and *SSS_k_* are two or three connected SMILES atoms. FLS means fragments of local symmetry [10], i.e., fragments of SMILES that can be represented as XYX, XYYX, or XYZYX, where X ≠ Y and Y ≠ Z. 

APP is the matrix of atom pair proportions [28]. *T* and *N* are parameters of the Monte Carlo optimization that provide numerical data on the correlation weights (CWs) for the SMILES attributes.

Models

The model generated by the CORAL software was calculated as

(2) $$p{IC}_{50}=C_{0}+C_{1}\times DCW(T,N)$$

*C*_0_ and *C*_1_ are regression coefficients.

Monte Carlo method

The Monte Carlo optimization provides the numerical data on the correlation weights of the SMILES attributes listed above. The following calculation aims to provide a larger value for target functions:

(3) $$T_{1}=RA+RP-\left|RA-RP\right|\times F_{1}+(IIC+CII)\times F_{2}$$

(4) $$T_{2}=RA+RP-\left|RA-RP\right|\times F_{1}+\left(IIC+CII\right)\times F_{2}+CCCP\times F_{3}$$

RA and RP are correlation coefficients for active and passive training sets, respectively; the index of ideality of correlation (IIC) [11]; the correlation intensity index [8]; and the coefficient of the conformism of a correlative prediction (CCCP) [8] are components of the Monte Carlo optimization—F_1_ = F_2_ = 0.5; F_3_ = 0.3.

Classification models

Database 2

The second database contains data on 13,846 organic molecules for classification models [17]. The descriptors are as we described above.

Models

A unique feature of the approach under consideration is the possibility of constructing so-called semi-correlations [11], which are tools for the representation of binary classifications based on the principle of active versus inactive, represented by 1 and 0 (in principle, the other option, namely active = +1 and inactive = −1, can be used too).

The use of semi-correlations is carried out by means of a regression model in which the values of the optimal descriptor calculated by Equation (1) are plotted along one axis (ordinate), and along the abscissa, there are only two values of 0 and 1 (or, as mentioned above, −1 and +1) for the identification of activity and non-activity, respectively. This binary classification is carried out according to the following scheme:

(5) $$y=C_{0}+C_{1}\times DCW(T,N)$$

(6) $$Classification\left(SMILES\right)=\left\{\begin{array}{c} 1\left(active\right),\;\;if\;y\geq 0.5\\ 0\left(inactive\right),\;if\;y<0.5 \end{array}\right.$$

Applicability domain

The modeling system, through the CORAL program, assumes a stochastic nature in several aspects. First, it is assumed that the model can be built for any random split in training and validation sets. It is expected that some distributions will lead to a good statistical quality of the model and some to a low statistical quality. Secondly, it is expected that even for “successful” models, there will be a scatter in the statistical characteristics (correlation coefficient and standard deviation). Thus, criteria are needed to select suitable distributions for training and validation. Statistical defect values for molecular features extracted from SMILES and statistical defect values for distributions have been proposed [10]. Depending on the statistical defects of the molecular features, as well as their average values, the applicability domain is determined, as shown below. 

The defects for SMILES features (which represent molecular features) are calculated as follows:

(7) $$d_{k}=\frac{\left|{P(A}_{k})-{P^{\prime }(A}_{k})\right|}{N\left(A_{k}\right)+N^{\prime }(A_{k})}+\frac{\left|{P(A}_{k})-{P^{\prime \prime }(A}_{k})\right|}{N\left(A_{k}\right)+N^{\prime \prime }(A_{k})}+\frac{\left|{P^{\prime }(A}_{k})-{P^{\prime \prime }(A}_{k})\right|}{N^{\prime }\left(A_{k}\right)+N^{\prime \prime }(A_{k})}$$

where P(A_k_), P′(A_k_), and P″(A_k_) are the probabilities of A_k_ in the active training, passive training, and calibration sets, respectively, and N(A_k_), N′(A_k_), and N″(A_k_) are the frequencies of A_k_ in the active training set, passive training set, and calibration set. The statistical SMILES defects (D_j_) are calculated as follows:

(8) $$D_{j}=\sum_{k=1}^{NA}d_{k}$$

where NA is the number of non-blocked SMILES attributes in the SMILES.

A SMILES falls into the domain of applicability if

(9) $$Dj<2*\bar{D}$$

$\bar{D}$ is average on the list of SMILES attribute defects {Dj}.

Las Vegas algorithm

The Las Vegas algorithm is a sequence of testing for different splits into active training, passive training, calibration, and validation sets in the process of the Monte Carlo optimizations [8]. The aim of the algorithm used here is the split which provides a determination coefficient for the calibration set that is as large as possible, hoping that it is accompanied by a large determination coefficient for the external validation set. Table 6 contains an example of the Las Vegas algorithm functioning. 

Mechanistic interpretation

Through the comparison of several starting points of the described Monte Carlo optimization under the same conditions (same split and same parametrization), one can select a group of SMILES attributes (i.e., the group of molecular features) with positive correlation weights. These can be considered promoters of increases for the endpoint under consideration. Table 7 contains a collection of molecular features with positive correlation weights in several areas of stochastic optimization. One can see that in the absence of fragments of local symmetry, XYYX and XYZYX are promoters of the increase in cardiac toxicity in hERG. The same role is present in certain atoms, such as nitrogen, and plays a significant part in aromaticity (Table 7). 

## 5. Conclusions

Cardiotoxicity models (hERG-blocking compounds expressed as pIC50, Database 1) are proposed based on the biological activity of 394 compounds, aiming to build regression models and providing results as continuous values. Furthermore, another set of compounds from Database 2, which contained 13864 compounds, was applied to develop a classification model. For both databases, good results were achieved. Stochastic Monte Carlo simulation algorithms for the different distribution of the substances into three training sets and a validation set were used. In addition, two functions were used to optimize the correlation weights of molecular features extracted from SMILES by utilizing CORAL software. Five main principles of model construction using CORAL software were formulated to simplify the understanding and possible application of the considered algorithms as much as possible. The proposed approach is convenient since it facilitates the study of possible effects of substances, providing the user with the opportunity to formulate and test various hypotheses related to cardiotoxicity simulation. In particular, the possibility of involving the described types of local symmetry in the simulation was tested in this study. In addition, the effectiveness of the new criterion of predictive potential (CCCP) was tested, and the effectiveness of IIC and CII as criteria of the predictive potential of models was confirmed. This series of optimization phases and approaches have shown to be effective in producing novel in silico models to be used to analyze endpoints related to cardiotoxicity. These novel models provided continuous or classification values, with statistical parameters, as good as or superior to those obtained with other models. An advantage of the CORAL software is that it does not require the calculation of molecular descriptors, and thus it is simpler and more convenient. 

## Figures and Tables

**Table 1:** Statistical characteristics of models obtained with target function T_1_.

	n *	R^2^	CCC	IIC	CII	Q^2^	CCCP	RMSE	MAE	F
A	101	0.660	0.795	0.765	0.794	0.648	0.198	0.802	0.599	192
P	96	0.690	0.748	0.655	0.812	0.679	0.331	0.896	0.737	208
C	101	0.762	0.862	0.873	0.893	0.746	0.673	0.510	0.392	318
V	96	0.660	-	-	-	-	-	0.60	0.47	-
A	100	0.530	0.690	0.594	0.747	0.508	0.008	0.938	0.761	109
P	99	0.565	0.703	0.523	0.745	0.549	−0.011	0.964	0.804	126
C	96	0.746	0.859	0.864	0.848	0.732	0.382	0.464	0.350	276
V	99	0.647	-	-	-	-	-	0.46	0.38	-
A	98	0.608	0.756	0.749	0.772	0.590	0.113	0.817	0.673	149
P	98	0.685	0.762	0.799	0.801	0.674	0.211	0.919	0.798	208
C	99	0.777	0.879	0.881	0.881	0.764	0.607	0.359	0.292	338
V	99	0.682	-	-	-	-	-	0.50	0.38	-

(*) n = number of substances.

**Table 2:** Statistical characteristics of models obtained with target function T_2_.

	n *	R^2^	CCC	IIC	CII	Q^2^	CCCP	RMSE	MAE	F
A	101	0.562	0.720	0.627	0.760	0.544	0.141	0.909	0.710	127
P	96	0.552	0.672	0.374	0.778	0.536	0.214	0.994	0.807	116
C	101	0.828	0.909	0.909	0.933	0.815	0.858	0.387	0.290	475
V	96	0.773	-	-	-	-	-	0.44	0.33	-
A	100	0.536	0.698	0.676	0.755	0.516	0.135	0.929	0.763	113
P	99	0.532	0.691	0.592	0.750	0.516	0.011	0.995	0.825	110
C	96	0.824	0.905	0.907	0.904	0.814	0.763	0.379	0.296	439
V	99	0.706	-	-	-	-	-	0.41	0.33	-
A	98	0.526	0.690	0.642	0.753	0.506	0.094	0.899	0.764	107
P	98	0.603	0.716	0.747	0.750	0.589	0.023	1.00	0.825	146
C	99	0.817	0.902	0.904	0.923	0.805	0.825	0.324	0.263	434
V	99	0.716	-	-	-	-	-	0.45	0.35	-

(*) n = number of substances.

**Table 3:** Comparison of the predictive potential for cardiotoxicity models.

n *	R^2^	Reference
400	0.52	[1]
137	0.81	[12]
400	0.52	[13]
529	0.59	[14]
345	0.41	[15]
840	0.66	[16]
394	0.64	Present study

(*) n = number of substances.

**Table 4:** The statistical characteristics of the model for cardiotoxicity are based on semi-correlations obtained using target functions T_1_ and T_2_.

Target Function	Split	Set *	Sensitivity	Specificity	Accuracy	Matthews Correlation Coefficient
T_1_	1	C	0.990	0.986	0.988	0.976
		V	0.990	0.990	0.990	0.979
	2	C	0.964	0.962	0.963	0.927
		V	0.968	0.967	0.967	0.934
	3	C	0.970	0.967	0.969	0.938
		V	0.980	0.965	0.972	0.945
T_2_	1	C	0.999	0.997	0.998	0.996
		V	1.000	0.998	0.999	0.998
	2	C	0.988	0.986	0.987	0.974
		V	0.992	0.990	0.991	0.982
	3	C	0.994	0.988	0.991	0.982
		V	0.996	0.988	0.992	0.983

(*) C = calibration set; V = validation set.

**Table 5:** Comparison of the statistical characteristics of classification cardiotoxicity models from the literature with models built using semi-correlative models obtained with CORAL software.

Accuracy	Sensitivity	Specificity	References
0.984	0.670	0.995	[17]
0.905	0.702	0.912	[19]
0.664	0.865	0.657	[20]
0.568	0.876	0.557	[21]
0.998	0.998	1.000	[22]
0.865	0.858	0.871	[23]
-	0.930	0.900	[24]
0.876	0.871	0.882	[25]
0.930	0.967	0.780	[26]
0.840	0.824	0.858	[27]
0.999	0.999	0.998	Split 1, target function T_2_
0.991	0.992	0.990	Split 2, target function T_2_
0.992	0.995	0.988	Split 3, target function T_2_

**Table 6:** An example of the Las Vegas algorithm features.

Test *	W%	N_111_	N_110_	N_101_	N_100_	N_All_	CCCP	R^2^_A_	R^2^_P_	R^2^_C_	Best R^2^_C_	Best Test
1	97	291	5	4	1	301	0.527	0.830	0.813	0.746	0.746	1
2	97	299	5	4	0	308	0.424	0.771	0.748	0.713	0.746	1
3	97	289	6	4	0	299	0.740	0.700	0.667	0.776	0.776	3
4	96	281	9	3	0	293	0.777	0.828	0.751	0.621	0.776	3
5	93	287	18	3	2	310	0.760	0.822	0.807	0.706	0.776	3
6	90	283	28	0	3	314	0.659	0.830	0.830	0.782	0.782	6
7	93	291	18	1	3	313	−0.113	0.480	0.461	0.000	0.782	6
8	95	283	12	2	1	298	0.624	0.765	0.769	0.569	0.782	6
9	96	288	11	1	0	300	0.655	0.796	0.795	0.548	0.782	6
10	91	287	21	4	3	315	0.671	0.830	0.820	0.768	0.782	6

(*) The test is a probe of the Monte Carlo optimization; W% = the percentage of the SMILES attributes taking part in the process of the Monte Carlo optimization; N_111_ = the number of SMILES attributes observed in active training, passive training, and calibration sets; N_110_ = the number of SMILES attributes observed in active and passive training sets (only); N_101_ = the number of SMILES attributes observed in the active training set and calibration set (only); N_100_ = the number of SMILES attributes observed in only the active training set; R^2^_A_, R^2^_P_, and R^2^_C_ are determination coefficients for the active training, passive training, and calibration sets, respectively.

**Table 7:** A collection of promoters of cardiotoxicity increases.

SMILES Attribute	CWs, Probe 1	CWs, Probe 2	CWs, Probe 3	CWs, Probe 4	CWs, Probe 5
c……	0.459	0.459	0.459	0.459	0.459
c…c……	0.084	0.146	0.146	0.146	0.146
C…(……	0.122	0.184	0.184	0.184	0.184
[xyyx0]…	1.760	0.384	0.384	0.384	0.384
c…(……	0.117	0.242	0.242	0.242	0.242
c…1……	1.289	0.351	0.351	0.351	0.351
c…c…c…	0.757	0.257	0.257	0.257	0.257
N……	0.075	0.137	0.137	0.137	0.137
c…c…1…	0.299	0.424	0.424	0.424	0.424
[xyzyx0]…	0.085	0.210	0.210	0.210	0.210
C…C……	0.101	0.351	0.351	0.351	0.351
O……	0.186	0.248	0.248	0.248	0.248
N…C……	0.259	0.321	0.321	0.321	0.321
N…(……	0.064	0.127	0.127	0.127	0.127
c…c…(…	0.064	0.439	0.439	0.439	0.439

## Data Availability

Data are available in the article and its Supplementary Materials.

## Supplementary Materials

The following supporting information can be downloaded at: https://www.mdpi.com/article/10.3390/toxics13040309/s1. Table S1: Technical details on split 1 (Database 1). Table S2: Technical details on experimental validation (for external 200 compounds). Table S3: Technical details on the classification model of cardiotoxicity for Database 2.
